# GAD65 Antibody ELISA With Extended Reportable Range: Validation and Guidance for Neurological Practice

**DOI:** 10.1002/acn3.70378

**Published:** 2026-03-28

**Authors:** Andrew McKeon, Dana Olofson, Diana Anissian, Divyanshu Dubey, Eoin P. Flanagan, Max C. Herman, Sarosh R. Irani, Daniel H. Lachance, Maria A. Willrich, Sean Pittock, John R. Mills, Anastasia Zekeridou

**Affiliations:** ^1^ Department of Laboratory Medicine and Pathology Mayo Clinic Rochester Minnesota USA; ^2^ Department of Neurology Mayo Clinic Rochester Minnesota USA; ^3^ Department of Neurology Mayo Clinic Jacksonville Florida USA; ^4^ Department of Neuroscience Mayo Clinic Jacksonville Florida USA

**Keywords:** ataxia, autoimmune, ELISA, GAD65, stiff‐person

## Abstract

**Objective:**

To (1) validate GAD65‐ELISA detection and quantification for type 1 diabetes mellitus and autoimmune neurological diagnoses, (2) correlate ELISA results (reference range < 5 IU/mL) with established radioimmunoprecipitation assay (RIA; ≤ 0.02 nmol/L), and (3) define ELISA clinical utility and pitfalls.

**Methods:**

Serum performance for diabetes (FDA‐cleared, undiluted) was verified, and neurological laboratory‐developed serum and CSF dilution protocols were validated to extend the reportable range beyond > 250 IU/mL. ELISA and RIA values were correlated, including established neurological RIA cut‐offs (serum ≥ 20 nmol/L; CSF any positive).

**Results:**

ELISA met analytical criteria (precision, accuracy, sensitivity, specificity, reference range) in serum and CSF and was clinically equivalent to RIA for autoimmune diabetes. Neurological ELISA cut‐offs were established at 10,000 IU/mL (serum) and 100 IU/mL (CSF). Precision was better below 10,000 IU/mL (CVs < 20%) than above (CVs 30.8% serum; 26.5% CSF). ELISA–RIA correlation and agreement was stronger below the neurological cut‐off (R^2^ = 0.89) than above (R^2^ = 0.36). Positive agreement for RIA‐defined neurological disease was 100% in serum and CSF; all serums were > 10,000 IU/mL. Clinical specificity was 97.5% in serum and 100% in CSF, exceeding reported RIA specificity. Screen results > 250 IU/mL spanned a wide range of dilution values; many were below the neurological cut‐off. Most patients with paired serum/CSF positivity showed elevated GAD65 IgG indices.

**Interpretation:**

GAD65 ELISA and RIA have equivalent sensitivity for autoimmune diabetes and neurological testing, with higher specificity for ELISA. A serum cut‐off of 10,000 IU/mL is informative but requires clinical context, and dilution of screen > 250 IU/mL samples is essential for neurological interpretation.

## 1 Introduction

Antibody targeting CNS‐ and endocrine pancreas‐enriched glutamic acid decarboxylase 65 kilodalton isoform (GAD65) is informative of both autoimmune type 1 diabetes and autoimmune CNS disease diagnoses (limbic encephalitis, epilepsy, ataxia, brainstem disorders, stiff‐person syndrome, and myelopathies) [1, 2, 3, 4, 5]. Serum testing aids type 1 diabetes and autoimmune CNS disease diagnostic testing; CSF testing is reserved for neurological disease [4].

For three decades, we have utilized a GAD65‐specific radioimmunoprecipitation assay (RIA) with ≥ 0.03 nmol/L positive cut‐off value for autoimmune type 1 diabetes in serum, and an additional clinical cut‐off of ≥ 20 nmol/L in serum for autoimmune CNS diagnoses (with any positive CSF result generally considered autoimmune CNS disease specific) [6, 7, 8]. Sensitivity of GAD65 antibody for type 1 diabetes by RIA and enzyme‐linked immunosorbent assay (ELISA) are both approximately 80% [9]. Sensitivity for type 1 diabetes is optimized further by combining with testing for other islet cell antibodies (IA‐2, insulin, and zinc transporter 8) [10]. GAD65 antibody is detected in serum of 0.7%–8% of the general healthy population, typically yielding low positive values [7, 11]. “High positive” GAD65 antibody value ≥ 20 nmol/L by RIA is the fourth‐most common neurological disease‐relevant antibody detected in the Mayo Clinic Neuroimmunology Laboratory. ELISA is commonly utilized by diagnostic laboratories, most commonly using the Kronus GAD65 antibody ELISA FDA‐cleared kit with reference range of > 5 international units per milliliter (IU/mL) [12]. An ELISA cut‐off of > 10,000 IU/mL for neurological autoimmunity has been proposed [13, 14].

In our neurological practice experience, misinterpretation of positive GAD65 antibody results is common, exacerbated by those inter‐laboratory methodologic and reference range differences. In addition, many GAD65 antibody positive screen results from the FDA‐cleared kit exceed the upper limit of quantitation (250 IU/mL) and thereby limit the neurological diagnostic interpretation. Extending the clinical reportable range of the GAD65 ELISA with a dilution scheme should provide opportunity for improved interpretation of positive results for neurological significance, for harmonization of testing practices across diagnostic laboratories, and enable retirement of a radioactive assay.

Herein, we report our analytical and clinical validation of a GAD65 antibody ELISA kit that includes a lab‐developed modification of the FDA‐approved method to additionally include a serum dilution protocol and CSF testing.

## 2 Methods

This study was approved by the Mayo Clinic Institutional Review Board. Consent was obtained from all patients or legally authorized representatives.

### 2.1 Assays

All testing was performed in duplicate, per manufacturer guideline and our longstanding practice for ELISA and RIA. The assay uses the “bridging” ELISA principle. GAD65 autoantibodies present in the patient sample form a divalent bridge between GAD65 coated on ELISA plate wells and liquid phase GAD65‐biotin. The ELISA kit (Kronus, Star, Idaho, USA) contained a pre‐coated 96 well plate, calibrators, controls, and all reagents required for the performance of the assay (concentrated wash buffer, GAD65‐biotin, streptavidin peroxidase, colorogenic substrate (3,3′,5,5′‐tetramethylbenzidine [TMB]), and stop solutions) [12]. Patient samples were serum and CSF (both used 25uL, undiluted). The GAD65‐biotin bound was quantitated by addition of streptavidin peroxidase and TMB with reading of the final absorbances at 450 nm. The absorbance of each well (directly proportional to the amount of antibody present) was expressed as optical density (OD). Calibrator material provided by the manufacturer had values expressed in semi‐quantitative arbitrary units (international units, IU/mL) and were at levels 5, 18, 35, 120, and 250 IU/mL. These data were used to generate a standard curve, Figure S1. Data was reduced using a cubic spline curve fit with a logarithmic x‐axis (concentration) and a linear y‐axis (OD). The IU/mL values for patient serum and CSF samples were calculated using the standard curve. Reference range was > 5 IU/mL.

Serum and CSF samples that resulted in a screening value > 250 IU/mL were further diluted using 20‐fold dilutions from 1:20–1:160,000 (1:20, 400, 8000, and 160,000; 10 mcL sample into 190 mcL diluent). Heat‐inactivated 10% normal goat serum (50 mL) in PBS with Tween (450 mL) was manufactured to be used as diluent for both serum and CSF assays. Dilutions that resulted in IU/mL values between 6 and 250 were multiplied by the dilution factor for a final IU/mL value up to 40,000,000 IU/mL. Results > 250 at 1:160000 were reported as > 40,000,000 IU/mL. If more than one dilution produced calculable results, the highest final “> 5 IU/mL” value was chosen, so as to avoid reporting inaccurately low results due to prozone effect (antibody excess preventing effective lattice formation). Synthetic CSF used for analytical sensitivity testing was manufactured by UTAK (#35100).

GAD65 antibody radioimmunoprecipitation assay was performed as previously described [7]. Briefly, iodine‐125–labeled recombinant human GAD65 (Kronus) was mixed with patient serum and incubated at room temperature for 3 h. Goat anti‐human IgG was then incubated for 1 h at 4°C to allow immune complexes to form. Complexes were pelleted and washed, and the gamma emission (counts per minute) of the final pelleted complexes were measured using a gamma counter. Background counts per minute were subtracted from patient results, and then the resulting counts per minute were extrapolated to nanomoles per liter using a reference with known quantities of iodine‐125–labeled antigen [7]. Reference range was ≤ 0.02 nmol/L. For samples with a screen value ≥ 25% of the high positive control, additional 10‐fold dilutions (1:10, 1:100, 1:1000, etc.) were performed to determine the highest nmol/L value.

To facilitate high‐throughput screening, all liquid measures and transfers for validation testing were accomplished using automated pipetting systems (Hamilton Company).

### 2.2 Samples, Patients and Controls

Pooled serum and CSF positive samples across the analytical measurable range (3 of each) and negative samples were manufactured for precision assays.

RIA GAD65 antibody positive disease group patients included: neurological disease (serums 24, CSF 20), diabetes serums 20. GAD65 autoimmune neurological disease cases (24 total) had been diagnosed by ≥ 1 of the co‐authors: stiff‐person syndrome, ataxia, epilepsy (8 each). For reference range, the manufacturer had tested, in the course of validation for FDA clearance, 120 healthy donor sera. Additional Mayo controls were: normal healthy donor serums used for clinical specificity (from 40 patients [20 adult, 20 pediatric] obtained from the Mayo Clinic Biobank) and waste clinical CSF used for clinical specificity and reference range (from 40 Mayo Clinic patients without autoimmune neurological diagnoses [40; 20 adult, 20 pediatric]). For analytical specificity (ability of the test to accurately identify GAD65 antibody and avoid measuring or reacting with other substances), testing included eight specimens (2 positive and 2 negative each of serum and CSF) spiked with hemolyzed blood, lipid or bilirubin, and 20 serums from patients with monoclonal gammopathies.

Serums of 10 patients from our clinical practice considered by referring providers to have “high positive” GAD65 antibody results reported by outside laboratories but did not have GAD65 neurological phenotypes on assessment in our Autoimmune Neurology Clinic were tested by both GAD65 RIA and ELISA (including dilutions).

Waste specimens (200 serums, 88 CSFs) without histories available from the clinical laboratory were also tested to determine: RIA versus ELISA correlation and compare serum and CSF ELISA values.

### 2.3 Statistics

Median and ranges for RIA (nmol/L) and ELISA (IU/mL) values were reported. Instrument performance, inter‐method values, and serum and CSF values were compared for correlation (R^2^) using Passing‐Bablok regression (with 95% confidence intervals) and for bias by Bland–Altman plots (with 95% limits of agreement), Analyze‐it for Excel. Precision of ELISA IU/mL values across and between assays was reported as coefficients of variance (CVs %), with < 20% expected [15]. Interassay concordance assessments were undertaken for diabetes and neurological cohorts. As a possible measure of intrathecal production of IgG, a GAD65 antibody‐specific index was calculated (CSF GAD65 value/serum GAD65 value divided by CSF albumin mg per dL/serum albumin mg per dL). An IgG index of > 0.66 has been considered supportive of intrathecal GAD65 antibody synthesis, particularly if greater than the general IgG index value [16]. Mayo Clinic's laboratory cut‐off for IgG index is ≤ 0.85. A GAD65 antibody specific index > 1.0, with value greater than the overall IgG index, is supportive of GAD65 antibody specific intrathecal synthesis.

## 3 Results

### 3.1 Analytical Validation

#### 3.1.1 Precision

To assess reproducibility within and between assays (precision), for both serum and CSF, five pools (3 positive samples [with values low positive, 50–200 IU/mL, medium positive, 300–500 IU/mL, and high positive 10,000–25,000 IU/mL] and two negatives [< 5 IU/mL]) were repeated 20 times in one plate (intra‐assay) and also 20 times across 20 unique assay runs that included two GAD65 ELISA kit manufacturer lots (inter‐assay), Table 1. All negative samples remained negative, and the low and mid‐positive samples remained positive and had intra‐ and inter‐assay CVs < 20%. Intra‐assay CVs were < 20% for high positive pools. Inter‐assay CVs were > 20% for high positive pools (36.2% and 22.4% for serum and CSF respectively), though all values remained > 10,000 IU/mL across precision assays (mean, 22,129 IU/mL for serum, and 13,183 IU/mL for CSF).

**TABLE 1:** GAD65 antibody ELISA intra‐ and inter‐assay precision (IU/mL).

Intra‐assay	Serum value			CSF value		
	Low	Medium	High	Low	Medium	High
Mean	68.7	387	18,455	114	307	11,751
SD	4.3	29.9	1462	7.8	35.0	1405
%CV	6.3	7.7	7.9	6.9	11.4	12.0
Inter‐assay	Low	Medium	High	Low	Medium	High
Mean	72.5	430	22,129	114	337	13,183
SD	7.52	75.3	8005	18.0	55.7	2949
%CV	10.4	17.5	36.2	15.8	16.5	22.4

#### 3.1.2 Accuracy

**FIGURE 1:**
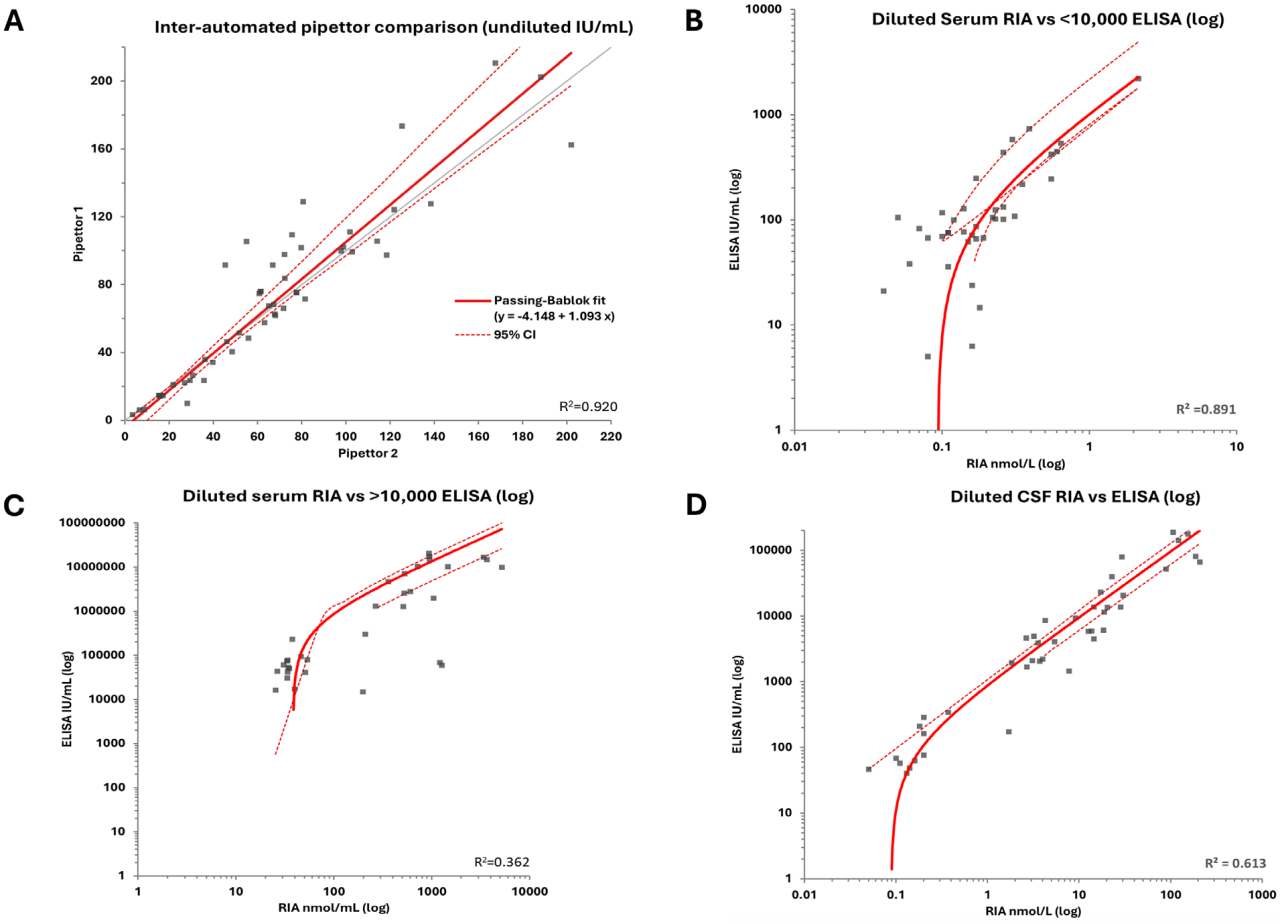
Passing‐Bablok regression analyses. Inter‐instrument comparison for automated liquid pipettors (A), log values for RIA (nmol/L) and ELISA (IU/mL) for lower positive serums (B), high positive serums (C), and CSF samples (D).

To assess accuracy (agreement compared to the gold standard measure) against RIA results, 49 serums (29 GAD65 RIA positive [18 with type 1 diabetes and 11 with neurological disease and RIA values ≥ 20 nmol/L] and 20 negatives) and 43 CSFs (23 GAD65 RIA positive and 20 negatives) were tested. All positive samples remained positive, and all negative samples remained negative. In addition, the high‐positive RIA serum samples all had values > 10,000 IU/mL. Inter‐instrument comparison of two automated liquid pipettors (Hamilton) using accuracy serum and CSF samples yielded concordant IU/mL values (R^2^ = 0.920, Figure 1A) with good agreement (Figure S2).

#### 3.1.3 Reference Range

Reference range refers to typical or normal results for a healthy population. Because positivity rates of up to 8% have been reported for GAD65 RIA in serum, the acceptance criterion for this study was 90% negative [7]. The manufacturer had previously tested 120 sera from healthy donors and reported 2% positivity (98% negative). Because large numbers of CSF specimens from normal healthy donors were not available to test, we tested 40 waste CSF specimens from patients with non‐autoimmune neurological disease diagnoses as surrogate, with an acceptance of 95% negativity rate. All were negative (≤ 5 IU/mL).

#### 3.1.4 Analytical Sensitivity

For detection capability (analytical sensitivity, or the ability of a test to accurately detect the smallest amount of GAD65 antibody in a sample), OD values for limit of blank (LoB), limit of detection (LoD), and IU/mL values for limit of quantification (LoQ) were calculated (results, Table S1). LoB was calculated by acquiring 20 measurements of a blank (healthy serum, synthetic CSF) tested in a single run. The mean and standard deviation of the ODs were calculated. LoB equaled the mean OD + 1.645 times the standard deviation of those blank measurements. LoD was calculated by acquiring 20 measurements of the lowest calibrator (5 IU/mL) tested in a single run. The mean and standard deviation of the ODs were calculated. LoD equaled the LoB plus 1.645 times the standard deviation of calibrator measurements. LoQ was calculated by acquiring 20 measurements of a low positive sample near the cutoff (> 5 IU/mL). The mean and % CV of the IU/mL were calculated (Table S1).

#### 3.1.5 Analytical Specificity

For analytical specificity (ability of the test to accurately identify GAD65 antibody specifically and avoid measuring or reacting with other substances in the sample), eight specimens (2 positive and 2 negative each of serum and CSF) were spiked with hemolyzed blood, lipid or bilirubin (Table S2). Hemolysis and lipemia did not interfere with testing (all had IU values within 20% of mock treated). However, positive CSF samples did not meet acceptance criteria at the highest concentration of icteric interference. Samples were spiked with decreasing concentrations of icteric interference (60 mg/dL to 3 mg/dL); the highest concentration that met criteria was 15 mg/dL. Thus, CSF samples that are visibly icteric (a very rare clinical scenario) were subsequently recommended to be rejected upon being received by the laboratory during clinical testing. Serums from 20 patients with potentially interfering monoclonal gammopathies were tested and were negative (≤ 5 IU/mL).

#### 3.1.6 Analytical Measuring Interval

To validate the extended range, positive samples (5 each of serum and CSF) across the Extended Measuring Interval (250–40,000,000 IU) were serially diluted in base matrix (negative sera or CSF), were tested as screens and reflexed to dilution. All samples met acceptance criteria (mean recovery between 80% and 120% for each sample), Table S3.

## 4 Clinical Validation

Reference range values of > 5 IU/mL and ≤ 0.02 nmol/L, respectively, were applied to define positive and negative for all analyses.

### 4.1 GAD65 Autoimmune Diabetes Mellitus Concordance Assessment

Serums from 39 patients with type 1 diabetes and known GAD65 antibody positivity by RIA were tested by ELISA and re‐tested by RIA. Median RIA value was 0.14 nmol/L (range, 0.06–2.14). Median ELISA value was 80.8 IU/mL (range, 5.03–2205).

All were positive by 1 or both assays: both assays (37), RIA only (1), or ELISA only (1). Positive percent agreement was 95%. Thus, both assays were 97.4% sensitive for known GAD65 autoimmune diabetes.

### 4.2 GAD65 Autoimmune Neurological Concordance Assessment

Equivalence of established RIA and the new ELISA methods to correctly identify patients with known GAD65 neurological autoimmunity was sought (clinical sensitivity). Serums from 24 patients with GAD65 neurological autoimmune diseases with known high value positivity by GAD65 RIA (defined as ≥ 20 nmol/L: median value, 266 nmol/L; range, 25.3–5179) were tested by ELISA and were positive; all had values ≥ 10,000 IU/mL (100% concordance); median value, 346,992 IU/mL; range, 14,934–19,117,120. CSF specimens from 20 patients with GAD65 autoimmune neurological disease with known positivity by GAD65 RIA (defined as ≥ 0.02 nmol/L: median value, 14.5 nmol/L; range, 0.37–208) were tested by ELISA and were positive (100% concordance): median value, 9770 IU/mL; range, 396–209,576.

### 4.3 Clinical Specificity

Forty serums from healthy donors and 40 CSFs from patients without immune mediated diagnoses were additionally tested and one serum was positive (serum, 97.5% specific; CSF 100% specific).

### 4.4 Data Derived From Diluting Samples With > 250 Values at Screening

Of 79 serums with a screen value of > 250 IU/mL reflexed for dilution during our pre‐validation and validation studies, 52 had a value of > 10,000 IU/mL (66%); median final value was 33,519 IU/mL (range, 275–41,000,960).

Serums from 10 patients referred to the Autoimmune Neurology Clinic at Mayo Clinic with GAD65 autoimmune neurological diagnosis based on outside laboratory ELISA values (in whom alternative neurological diagnoses were made at Mayo) were tested by RIA during clinical service and subsequently by ELISA in house (including dilution, Table 2). Eight of 10 were women; median age was 50 years, range 20–85. In half of those cases, a value of “> 250 IU/mL” had been reported by the outside laboratory yet the highest value in that cohort on follow‐up testing was 0.38 nmol/L by RIA and 4983 IU/mL by ELISA after dilution (thus, well below the expected threshold for autoimmune neurological diagnosis). Causes for GAD65 antibody positivity (other than autoimmune neurological disease) were encountered in five patients: type 1 diabetes in two; IVIg therapy prior to antibody testing in 3.

**TABLE 2:** Diluted and back calculated serum GAD65 antibody ELISA values, and RIA values and clinical data for 10 patients with erroneous GAD65 neurological diagnoses.

Patient	Outside diagnosis	Mayo diagnosis	Autoimmune diagnosis	IVIg prior to GAD65 Ab test	Outside GAD65 Ab ELISA reported value (IU/mL)	Mayo GAD65 Ab RIA value (nmol/L)	Mayo GAD65 Ab ELISA value (IU/mL)
1	SPS	Autoimmune chorea	None	Yes	> 250	0.12	179
2	SPS	Arthralgias, myalgias	None	No	> 250	0.45	503
3	GAD autoimmune disease	Dysarthria	None	No	> 250	0.75	242
4	SPS	Back and leg pains	None	No	> 250	0.22	142
5	SPS	Peripheral neuropathy	Type 1 diabetes, low B12	No	> 250	0.38	4983
6	SPS, LE	Arthralgias, depression	None	Yes	230	0.16	205
7	SPS	Non‐specific symptoms; normal examination	Type 1 diabetes, hypothyroid	No	128	0.03	< 5
8	SPS	HSP	None	No	128	0.24	144
9	SPS	Fibromyalgia	None	No	111.7	0.10	61.7
10	SPS	PD	None	Yes	60	0.14	84.6

Abbreviations: HSP, hereditary spastic paraplegia; LE, limbic encephalitis; PD, Parkinson disease; SPS, stiff‐person syndrome.

### 4.5 Autoimmune Diabetes Evaluation and Encephalitis Evaluation Serum Testing Requests

Seventy‐six waste serums from the clinical laboratory, a mixture of RIA positives (across the range of values) and negatives were evaluated. Thirty‐seven serums had been referred for diabetes mellitus evaluation. Median positive RIA value was 0.19 nmol/L (range, 0.04–1.16), and median positive ELISA value was 57.0 IU/mL (range, 5.41–> 250). Nineteen were positive by both assays (17), RIA only (1) or ELISA only (1), 95% agreement. Thirty‐nine had been referred for autoimmune encephalitis evaluation. Median positive RIA value was 0.10 nmol/L (range, 0.04–193), and median positive ELISA value was 32 IU/mL (range, 6–> 250). Of the 19 that were RIA positive, 8 were ELISA positive (42%). None were positive by ELISA only (overall, 72% agreement). The discrepant RIA positive/ELISA negative samples were all low positive by RIA (≤ 0.24 nmol/L), below the neurological cut‐off of 20 nmol/L supportive of higher ELISA specificity in this context.

### 4.6 Correlation of RIA and ELISA Values

RIA and ELISA values were assessed for correlation across the reportable range (Figure 1B,C). Included serums were 39 low positive serum samples (median ELISA value, 99.8 IU/mL; range, 5.03–2205; median RIA value, 0.66 nmol/L; range, 0.04–2.14) and 35 high positive serum samples (median ELISA value, 301,894 IU/mL; range, 14,866 – > 40,000,000: median RIA value, 359 nmol/L; range, 25.3–5179). Overall, there was stronger correlation for low positive serums (R^2^ = 0.891) than for high positive serums (R^2^ = 0.362), Figure 1B,C, and weak inter‐assay agreement at higher antibody values, Figure S2B,C.

Thirty‐eight serums spanning the range of RIA neurological cut‐off of 20.0 nmol/L (median value, 20.4 nmol/L; range, 12.6–30.4) were also tested by ELISA, and diluted as appropriate. Median ELISA value was 15,701 IU/mL (range, 3767–71,576). Correlation between those RIA and ELISA values was weak (R^2^ = 0.051). Thus, additional precision data were acquired for six serums with RIA values approximate to proposed “neurological cut‐off” for RIA (20 nmol/L; range 15.4–29.3), Table 3. Each of the six samples was tested six times by ELISA. Median values for the six serums ranged from 10,754 to 12,735 IU/mL. Median % CV was 28% (10%–39%). Notably, some replicates for the two serums with RIA values > 20 nmol/L had ELISA values < 10,000 IU/mL.

**TABLE 3:** Serums with GAD65 RIA values close to the neurological cut‐off of 20.0 nmol/L, their ELISA IU/mL values across 6 assays and inter‐assay precision (coefficient of variance [%CV]).

Serum	RIA	ELISA Run 1	ELISA Run 2	ELISA Run 3	ELISA Run 4	ELISA Run 5	ELISA Run 6	Median	%CV
1	29.3	8624	13,288	10,436	10,797	12,165	11,706	11,251	10
2	21.7	6840	11,147	10,626	7042	12,181	19,963	10,886	39
3	17.1	10,069	14,217	9729	15,216	12,897	11,810	12,353	17
4	17.0	10,016	20,342	12,790	11,177	11,545	12,680	12,735	28
5	16.3	9324	20,415	14,384	11,742	11,192	7225	11,467	38
6	15.4	10,666	13,613	8531	8072	10,842	11,515	10,754	22

For CSF, 42 samples with RIA values across the full reportable range were assessed. All were positive by ELISA, and had a median value of 4638 IU/mL (range, 40.5–189,016) and median RIA value of 4.26 nmol/L (range, 0.05–208), R^2^, 0.613, Figure 1D, and good interassay agreement (Figure S2D).

### 4.7 Correlation of Serum Versus CSF ELISA Values

**FIGURE 2:**
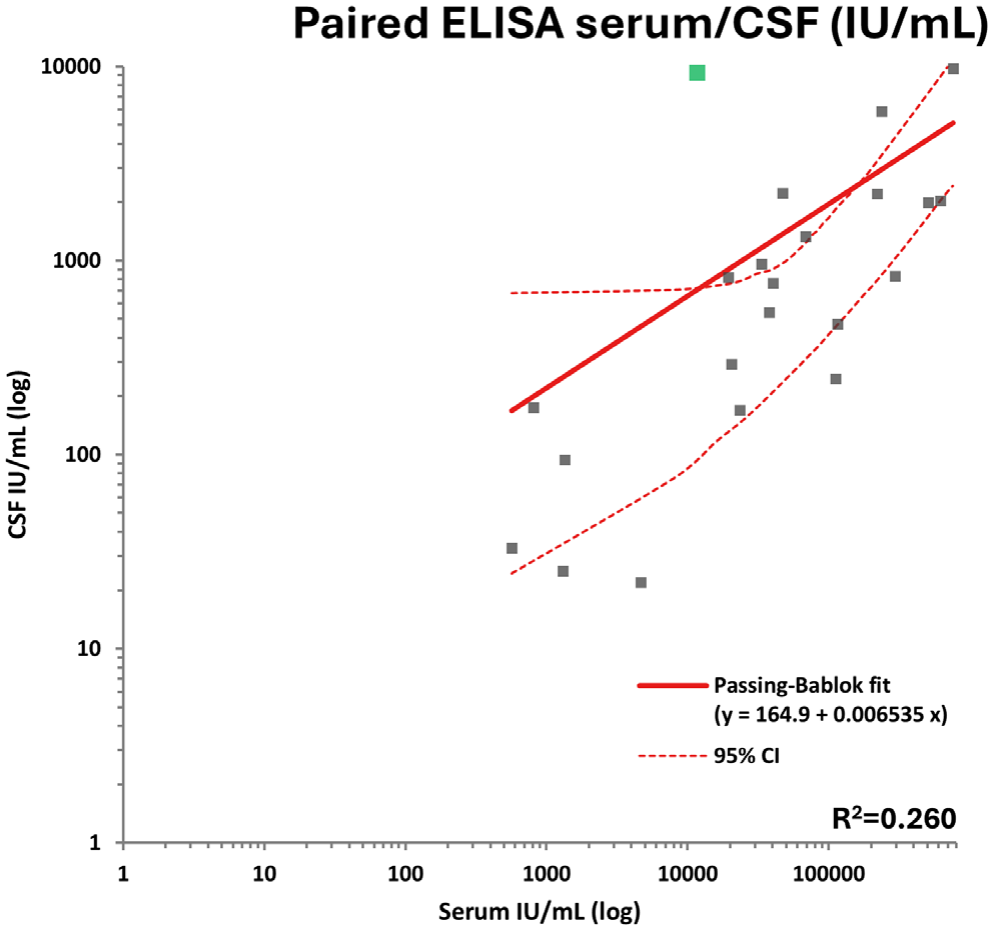
Correlation for log ELISA values for paired serum and CSF (E).

ELISA testing was also performed for 45 patients with paired serum and CSF waste samples available where at least one sample had been RIA positive; clinical histories were not available. Median serum ELISA value was 1302 IU/mL (range, 5.42–759,000); median CSF value was 33.1 IU/mL (range, ≤ 5–9714). The correlation was weak (R^2^ = 0.260; or 0.545 with removal of a single outlier, in green, Figure 2), though with good interassay agreement (Figure S3). An additional single patient had a negative serum value of ≤ 5 IU/mL (with RIA value of 0.03 nmol/L), and a positive CSF value of 1499 IU/mL (and RIA value of 1.08 nmol/L). The CSF and serum were drawn one month apart, and the patient had received no immunotherapy in between sample draws. Otherwise, the lowest serum value we encountered with a paired positive CSF was 567 IU/mL (Table S4).

### 4.8 GAD65 Antibody Specific Index

The GAD65 antibody index by ELISA was calculable in 15 patients with positivity of GAD65 antibody in both serum and CSF, and available albumin and overall IgG index data. Clinical information was available in seven of those patients (Table S4). The GAD65 index was positive in 11 of 15 patients and borderline in 1. Two of the seven patients with clinical information had a GAD65 antibody index of < 1.0; diagnoses were stiff‐person syndrome, 1; cerebellar ataxia, 1. The latter patient with a history of type 1 diabetes that started in his teens developed cerebellar ataxia in his 7th decade, within 1 month of starting pembrolizumab therapy for bladder urothelial carcinoma. The GAD65 index by RIA produced similar results to the ELISA; it was positive in 9 of 14 patients with data available, and borderline in 2 (Table S4).

## 5 Discussion

We report validation of the ELISA assay for GAD65 antibody, which has importance for the diagnosis of autoimmune type 1 diabetes and autoimmune CNS disease. Sensitivity and specificity performance of both ELISA and RIA GAD65 assays for type 1 diabetes is well established, and we confirmed equivalent performance of those assays for that diagnosis [17].

In this study our principal goal was to clinically validate the GAD65 antibody ELISA test for autoimmune neurological disease diagnoses in serum and CSF, including a dilution scheme to extend the clinical reportable range, and to evaluate for an approximate neurological clinical cut‐off in serum and CSF. We also report our analytical validation of this assay to ensure it met rigorous standards for accuracy, precision, reference range, reportable range, and analytical and clinical sensitivity and specificity, meeting the College of American Pathologist (CAP) requirements in accordance with Clinical Laboratory Improvement Amendments (CLIA) regulations from the Centers for Medicare & Medicaid Services (CMS), which regulates all human clinical laboratory testing in the United States. In addition, we adhere to the strict regulations required by the New York State Department of Health. As the serum test was already FDA‐cleared, we verified the analytical performance specifications provided by the manufacturer. To extend the clinical utility of the test for those with GAD65 neurological autoimmunity, we performed laboratory‐developed modifications, namely, testing in CSF and a dilution scheme to extend the clinical reportable range beyond 250 IU/mL in both serum and CSF. ELISA method in CSF matrix has been validated previously [18].

With neurological cut‐offs, we determined that the ELISA method had comparable concordance and specificity to RIA for GAD65 autoimmune neurological phenotypes. For those diagnoses, we have observed over time that patients with characteristic GAD65 autoimmune neurological phenotypes (stiff‐person syndrome, cerebellar ataxia, limbic encephalitis, myelopathy, or epilepsy) typically have GAD65 antibody RIA values in the 100 s of nmol/L, with lower end of values approximating 20 nmol/L. [8] An ELISA neurological cut‐off of 10,000 IU/mL by ELISA has been previously recommended [13, 14, 19]. Consistent with those prior reports we found 10,000 IU/mL by ELISA in serum to be approximately equivalent to a cut‐off of 20 nmol/L by RIA. All patients in our neurological cohort with GAD65 antibody serum RIA values of ≥ 25.0 nmol/L had an ELISA value of ≥ 14,000 IU/mL. Evaluating serum samples approximating that RIA cut‐off of 20 nmol/L (15–29 nmol/L), median GAD65 ELISA values ranged from 10,000 to 12,000, though occasional replicates in our precision study were as low as 7000–8000. Therefore, particularly close scrutiny for clinical correlation of results between 7000 and 12,000 IU/mL is advised.

We have observed over time that GAD65 antibody positivity in CSF correlates to some extent with serum values, though with lower values than in serum, and does not confer additional diagnostic specificity over serum testing. In this study, ELISA values of paired serum and CSF samples had limited correlation, though any serum value > 10,000 IU/mL had a corresponding CSF value of at least 100 IU/mL. We also evaluated a GAD65 serum/CSF index, which supported evidence of GAD65 antibody intrathecal synthesis in 11 of 15 patients with GAD65 antibody positivity in serum and CSF. For seven of those with histories available, five had GAD65 antibody positivity in serum and CSF (index > 1.0). For the remaining two cases, although serum values were > 10,000 IU/mL and CSF GAD65 antibody was also positive, the GAD antibody index was negative. One patient had typical stiff‐person syndrome. Consistent with prior reports, the index can be low or borderline in some stiff‐person syndrome patients [16]. The remaining patient had an unusual presentation: in the context of longstanding autoimmune diabetes and contemporaneous checkpoint inhibitor therapy for cancer, an exacerbation of peripherally‐generated GAD65 autoimmunity seemingly occurred, resulting in cerebellar ataxia. GAD65 neurological autoimmunity has been previously reported to occur during checkpoint inhibitor therapy [20, 21]. GAD65 index by RIA revealed similar findings to the ELISA index.

In general, for low positive GAD65 ELISA serum results, inter‐ and intra‐assay precision and correlation with RIA results were high (R^2^ = 0.89). However, for GAD65 samples requiring further dilutions to acquire the final value, inter‐ and intra‐assay precision and correlation and agreement with RIA results were lower. In our experience, lack of precision has also been evident for “high positive” RIA results. Given the imprecision at high positive values, the most pertinent interpretation is that the result is indeed “high,” using the neurological clinical cut‐off of approximately ≥ 10,000 IU/mL to adjudicate likelihood of GAD65 autoimmune neurological diagnosis. We do not recommend using longitudinal GAD65 antibody values (particularly across assay methods) as a surrogate of clinical response to treatment or prognosis [22].

We also found that an ELISA GAD65 serum antibody value of > 250 IU/mL at screening in a neurological context was not informative as a surrogate for a dilution scheme with end‐point value (a back calculated IU/mL based on the first dilution that falls within the analytical measurement range). Upon dilution of these “> 250” samples, results spanned a wide range of values (275 to 40 million IU/mL; median 33,519), one‐third of which had final values < 10,000 IU/mL. In our experience, many patients with GAD65 antibody positivity are misdiagnosed with an autoimmune neurological disease on the basis of symptomatology alone, and any GAD65 antibody positive result irrespective of titer. The “> 250” value (or indeed lower values) may be misleading or misinterpreted. In our clinical cohort of “real‐life” erroneous GAD65 autoimmune neurological diagnoses, referred to our subspecialty clinic, values of > 250 IU/mL were not replicated as “high positive” results by RIA (done in “real time”) or by ELISA with dilutions (in the course of this study). We previously reported that mimics of stiff‐person syndrome account for 72% of patients whom we are asked to assess for that diagnosis in our clinical practice [23]. Of 125 patients ultimately diagnosed with a stiff‐person syndrome mimic, 67 (54%) had GAD65 antibody values in serum below the neurological clinical cut‐off (20 nmol/L by RIA). Notwithstanding that, a further six mimics had GAD65 antibody value above that cut‐off, and one was CSF positive. In our laboratory and clinical experience, GAD65 antibody results above the neurological cut‐off are sometimes encountered in patients with type 1 diabetes without neurological symptoms or in patients with non‐autoimmune neurological diagnoses [13]. Thus, GAD65 antibody positivity and values need to be carefully interpreted in the context of the patient history and examination.

GAD65 antibody positivity is common in the general population (2% by ELISA in both this and prior ELISA kit manufacturer studies, and 8% by RIA), and even more common in those with type 1 diabetes, autoimmune thyroid disease and pernicious anemia, without CNS disease, typically at low positive values [7]. In addition, using RIA, we have observed frequent false low positives among patients referred for non‐diabetic indications (e.g., encephalopathy, movement disorders). In this study, the high level of RIA versus ELISA agreement for diabetic patient samples and low agreement for encephalopathy patient samples were both notable outcomes. The lower frequency of low positive GAD65 results by ELISA in the general population and among patients tested in the setting of altered mental status (most of whom do not have encephalitis) likely reflects better specificity for the ELISA than the RIA.

In conclusion, this GAD65 antibody ELISA is analytically robust and largely equivalent to our established RIA for diagnostic purposes for autoimmune diabetic and neurological phenotypes, though ELISA specificity appears to be better among neurological referrals. In addition, extending the clinical reportable range, particularly in serum, aids in differentiation of non‐neurological autoimmunity cases from true GAD65 autoimmunity where screen values are > 250 IU/mL. A neurological cut‐off in serum of approximately 10,000 IU/mL is useful, though occasional GAD65 autoimmune neurological patients have lower values but close to this level (> 7000 IU/mL). CSF positivity > 100 IU/mL is typical for GAD65 autoimmune neurological patients. We emphasize the importance of careful assessment of neurological phenotype and correlation with the antibody test result. Although “high positive” values tend to remain high upon repeat testing, there is low precision for numerical values which would limit prognostic interpretation in longitudinal studies. GAD65 antibody index may be pathophysiologically informative.

## Author Contributions

A.M., D.O. and J.R.M. study design. All authors: data acquisition. A.M., D.O., J.R.M. and A.Z. data analysis and interpretation. A.M. and D.O. manuscript preparation. All authors: Critical revision of the manuscript for intellectual content. A.M. study supervision and funding.

## Funding

This work was supported by the National Institute of Neurological Disorders and Stroke (Grant R01 NS126227).

## Conflicts of Interest

The authors declare no conflicts of interest.

## Supporting information


**Figure S1:** Standard curve for GAD65 ELISA to generate IU/mL values from OD values.

**Figure S2:** For GAD65‐IgG antibody testing, Bland–Altman (Tukey difference) plots were used to assess for agreement between: two automated liquid handlers for GAD65‐IgG ELISA testing (A), RIA versus ELISA in serum (B, lower ELISA values; and C, higher ELISA values), and CSF (D). The average difference is represented by the solid blue line and the 95% limits of agreement (LoAs) by the broken blue lines. Agreement was acceptable overall, though the difference between RIA and ELISA in serum (B and C) increased with the magnitude of the measurement.

**Figure S3:** For GAD65‐IgG ELISA antibody testing, a Bland–Altman (Tukey difference) plot was used to assess for agreement between measurements in individual patient serum–CSF pairs. The average difference (bias) is represented by the solid blue line and the 95% limits of agreement (LoAs) by the broken blue lines. There was clustering of data points close to the average difference indicative of strong agreement.

**Table S1:** Detection capability (analytical sensitivity).
**Table S2:** Interference studies.
**Table S3:** Extended measuring interval validation.
**Table S4:** Serum & CSF GAD65 ELISA antibody values, albumin data, IgG & GAD65 antibody indices for 15 patients, with clinical treatment and outcome data (where available).

## Data Availability

Data are available from the corresponding author upon reasonable request.
