# Supplementary figures and images for: GAD65 Antibody ELISA With Extended Reportable Range: Validation and Guidance for Neurological Practice
Source: Ann Clin Transl Neurol. 2026 Mar 28:10.1002/acn3.70378. Online ahead of print. doi: 10.1002/acn3.70378 (PMC13394602; doi:10.1002/acn3.70378)

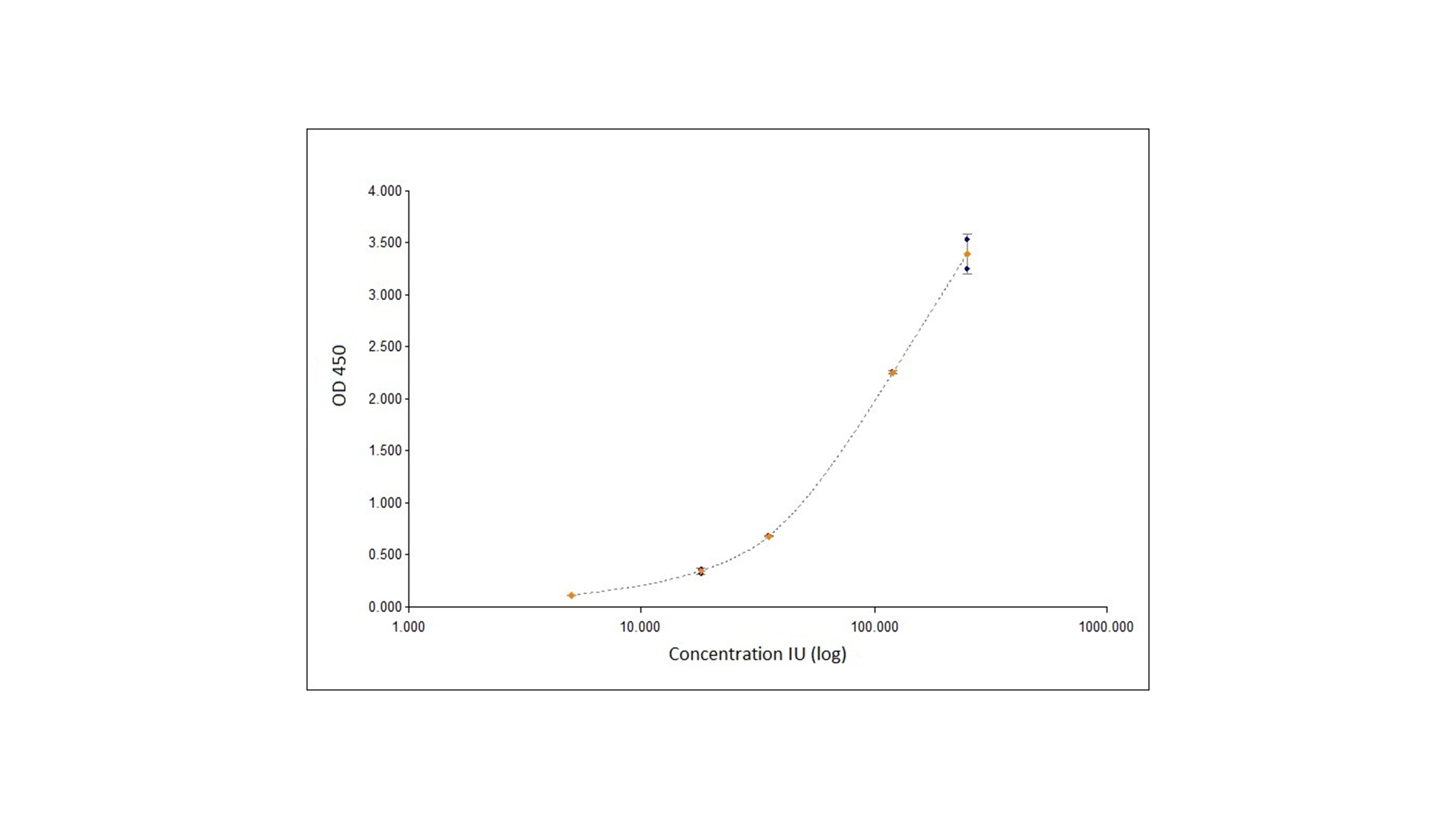

Supplement: Supplementary file 1 — Figure S1: Standard curve for GAD65 ELISA to generate IU/mL values from OD values. [file ACN3-9999-0-s002.tif]

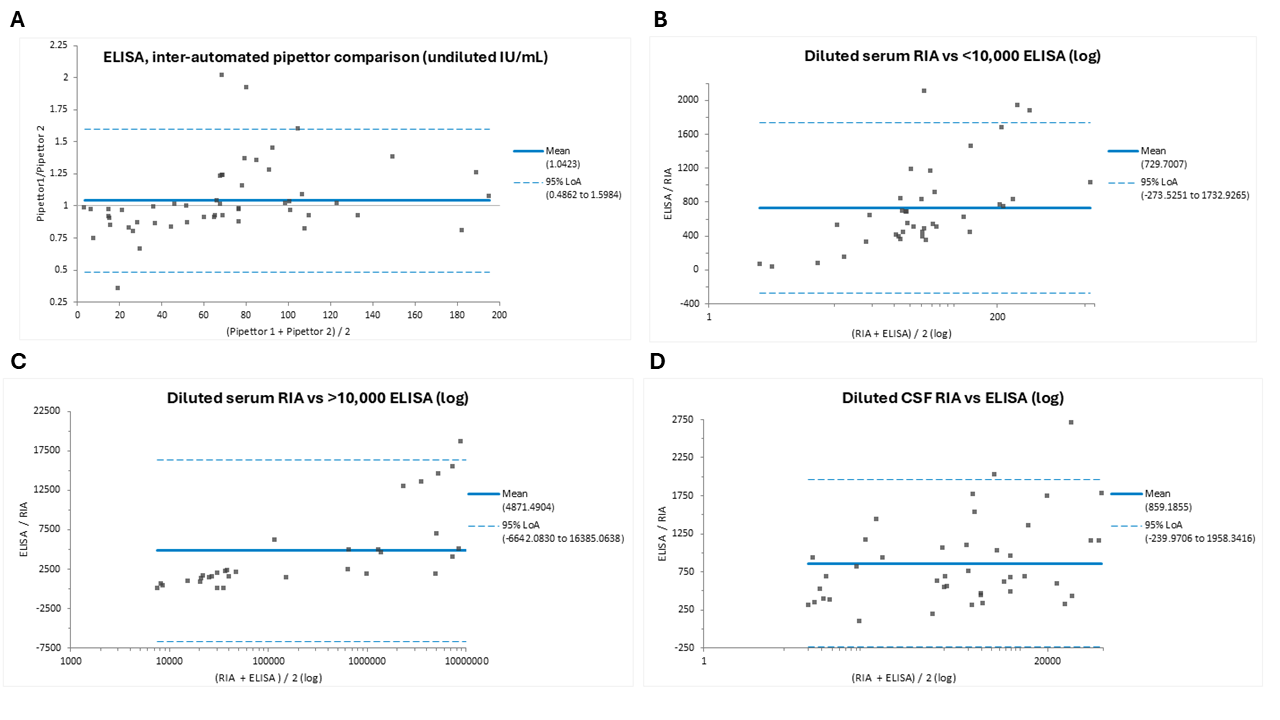

Supplement: Supplementary file 2 — Figure S2: For GAD65‐IgG antibody testing, Bland–Altman (Tukey difference) plots were used to assess for agreement between: two automated liquid handlers for GAD65‐IgG ELISA testing (A), RIA versus ELISA in serum (B, lower ELISA values; and C, higher ELISA values), and CSF (D). The average difference is represented by the solid blue line and the 95% limits of agreement (LoAs) by the broken blue lines. Agreement was acceptable overall, though the difference between RIA and ELISA in serum (B and C) increased with the magnitude of the measurement. [file ACN3-9999-0-s001.tif]

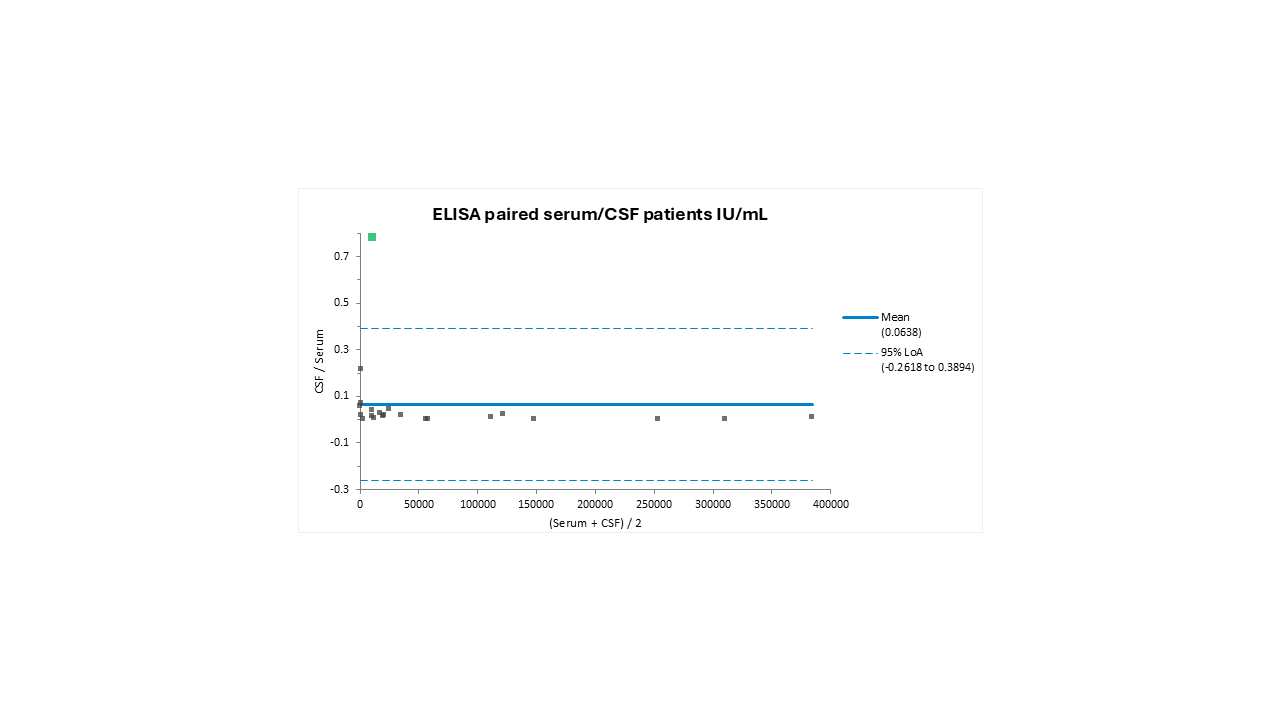

Supplement: Supplementary file 3 — Figure S3: For GAD65‐IgG ELISA antibody testing, a Bland–Altman (Tukey difference) plot was used to assess for agreement between measurements in individual patient serum–CSF pairs. The average difference (bias) is represented by the solid blue line and the 95% limits of agreement (LoAs) by the broken blue lines. There was clustering of data points close to the average difference indicative of strong agreement. [file ACN3-9999-0-s003.tif]
