# Supplementary material for: GAD65 Antibody ELISA With Extended Reportable Range: Validation and Guidance for Neurological Practice
Source: Ann Clin Transl Neurol. 2026 Mar 28:10.1002/acn3.70378. Online ahead of print. doi: 10.1002/acn3.70378 (PMC13394602; doi:10.1002/acn3.70378)
Supplement: Supplementary file 4 — Table S1: Detection capability (analytical sensitivity). Table S2: Interference studies. Table S3: Extended measuring interval validation. Table S4: Serum & CSF GAD65 ELISA antibody values, albumin data, IgG & GAD65 antibody indices for 15 patients, with clinical treatment and outcome data (where available). [file ACN3-9999-0-s004.docx]

**Supplementary Table 1**. Detection capability (analytical sensitivity)

|  | Serum | CSF |
| --- | --- | --- |
| LoB (OD) | 0.018 | 0.006 |
| LoD (OD) | 0.045 | 0.021 |
| LoQ (mean IU/mL) | 6.54 | 8.79 |
| LoQ %CV | 6.3% | 3.6% |

LoB = limit of blank; LoD = limit of detection; LoQ = limit of quantification; OD = optical density.

**Supplementary Table 2**. Interference studies

|  | **Serum** | | | | **CSF** | | | |
| --- | --- | --- | --- | --- | --- | --- | --- | --- |
|  | **Low**  **(% Change)** | **Mid**  **(% Change)** | **Neg 1 (IU)** | **Neg 2 (IU)** | **Low**  **(% Change)** | **Mid**  **(% Change)** | **Neg 1 (IU)** | **Neg 2 (IU)** |
| **Hemolysed blood**  **1,000 mg/dL** | 4.9% | 3.8% | <5 | <5 | -3.1% | 2.9% | <5 | <5 |
| **Lipid**  **2,000 mg/dL** | -5.9% | -6.2% | <5 | <5 | -13.6% | -10.6% | <5 | <5 |
| **Bilirubin**  **60 mg/dL** | 3.0% | -12.1% | <5 | <5 | 22.8%* | -56.5%* | <5 | <5 |
| **Bilirubin**  **15 mg/dL** |  |  |  |  | 10.2% | -18.4% |  |  |

Low = low positive GAD65 cohort. Mid = medium range positive GAD65.

**Supplementary Table 3.** Extended measuring interval validation

| **Specimen** | **Final IU** | **Mean recovery** | **Specimen** | **Final IU** | **Mean recovery** |
| --- | --- | --- | --- | --- | --- |
| Serum 1 | 359 | 102% | CSF 1 | 453 | 93% |
| Serum 2 | 4,925 | 100% | CSF 2 | 3,001 | 93% |
| Serum 3 | 15,927 | 98% | CSF 3 | 7,284 | 100% |
| Serum 4 | 106,168 | 101% | CSF 4 | 84,256 | 103% |
| Serum 5 | 4,774,507 | 102% | CSF 5 | 100,488 | 97% |

**Supplementary** **Table 4.** Serum & CSF GAD65 ELISA antibody values, albumin data, IgG & GAD65 antibody indices for 15 patients, with clinical treatment & outcome data (where available)

| **Pt** | **ELISA (IU/mL)**  **Serum, CSF** | **RIA**  **(nmol/L)**  **Serum, CSF** | **Albumin**  **(mg/dL) Serum, CSF** | **IgG Index CSF** | **GAD65 Index, ELISA** | **GAD65 Index, RIA** | **DM** | **Neurological diagnosis** | **Immune Treatment** | **Treatment outcome** |
| --- | --- | --- | --- | --- | --- | --- | --- | --- | --- | --- |
| 1 | 505000, 1981 | 398  0.00 | 4400  49.3 | 0.48 | 0.35 (N) | 0.00 (N) | Yes | Stiff limb syndrome | Steroids, rituximab | Improvement with steroids, rituximab. |
| 2 | 112407  254.28 | 242  0.48 | 4700  24 | 0.5 | 0.43 (N) | 0.39 (N) | NA | NA | NA | NA |
| 3 | 4661.2  21.93 | 4.28  0.04 | 4300  35.7 | 0.46 | 0.57 (N) | 1.13 (P) | NA | NA | NA | NA |
| 4 | 20412  293.73 | 29.9  0.44 | 3800  63.6 | 0.57 | 0.86 (B) | 0.88 (B) | Yes | Checkpoint inhibitor induced cerebellitis | Steroids, IVIg, PLEX, rituximab | Slow deterioration, bladder cancer. |
| 5 | 295000  833 | 356  0.65 | 4500  11.7 | 0.75 | 1.09 (P) | 0.70 (N) | Yes | Classical stiff person syndrome | IVIg, rituximab | Partial improvement |
| 6 | 759000  9714 | 782  110 | 4700  55.2 | 0.63 | 1.09 (P) | 11.98 (P) | Yes | Cerebellar ataxia, stiff limbs, seizures | PLEX, IV cyclophosphamide, mycophenolate | Partial improvement with PLEX & IV cyclophosphamide. Stable with mycophenolate |
| 7 | 567.28  33.07 | 0.22  0.06 | 2800  139.8 | 1.71 | 1.17 (P) | 5.46 (P) | NA | NA | NA | NA |
| 8 | 46944  2229.57 | 22.0  2.90 | 3400  123.3 | 1.27 | 1.31 (P) | 3.63 (P) | NA | NA | NA | NA |
| 9 | 68600  1328 | 163  1.50 | 3300  33.6 | 0.45 | 1.90 (P) | 0.90 (B) | Yes | Encephalitis, Ataxia | IVIg, oral cyclophosphamide, mycophenolate | Improved with IVIg |
| 10 | 33519  963 | 22.3  1.77 | 4600  35.7 | 0.71 | 3.70 (P) | 10.23 (P) | No | Limbic and potential brainstem encephalitis | Steroids | Stable with limited improvement |
| 11 | 40279  765.5 | 40.6  0.85 | 3400  16.8 | 0.64 | 3.85 (P) | 4.24 (P) | NA | NA | NA | NA |
| 12 | 1339.48  94.31 | 3.26  0.33 | 5200  48.9 | 0.5 | 7.49 (P) | 10.76 (P) | NA | NA | NA | NA |
| 13 | 38000  542 | 48.0  1.86 | 4100  5.4 | 0.92 | 10.83 (P) | 29.42 (P) | No | Autoimmune encephalitis (GABA_A_R & GAD65 Abs seropositive) | IVIG, steroids, PLEX, Rituximab | Full recovery, then relapsed after a year. Remission after repeat rituximab therapy. |
| 14 | 11799.2  9193.6 | NA  4.54 | 1100  12.6 | 0.38 | 68.02 (P) | NA | NA | NA | NA | NA |
| 15 | 811.66  174.72 | 2.70  0.51 | 4600  11.1 | 0.64 | 89.21 (P) | 78.28 (P) | NA | NA | NA | NA |

B = borderline; DM = diabetes mellitus; GAD65 = glutamic acid decaroxylase, 65 kilodlaton isoform; GABA_A_R = gamma amino butyric acid A receptor; IVIg= intravenous immue globulin; N = negative; NA = not available; P = positive; PLEX = plasma exchange; Pt = patient number.
